# Anaesthetic Challenges During Colonoscopy-Induced Intestinal Perforation in a Cat

**DOI:** 10.3390/vetsci13070707

**Published:** 2026-07-19

**Authors:** Dany Elzahaby, Bérénice Lutz, Isabelle Iff

**Affiliations:** 1 Vetsuisse Faculty, Department of Clinical Veterinary Medicine, Section of Anesthesiology and Pain Therapy, University of Bern, 3012 Bern, Switzerland; 2 School of Veterinary Medicine, Murdoch University, Perth, WA 6150, Australia; 3 Vetsuisse Faculty, Department of Clinical Veterinary Medicine, Section of Small Animal Internal Medicine, University of Bern, 3012 Bern, Switzerland; 4 Bessy’s Small Animal Clinic, IVC Evidensia, 8105 Regensdorf, Switzerland; 5 Vet Doc Iff GmbH, 4600 Olten, Switzerland

**Keywords:** colonoscopy, perforation, cat, embolism, pneumoretroperitoneum

## Abstract

While colonoscopies are generally benign procedures, rare complications such as colonic perforation may result in life-threatening consequences. A 15-year-old neutered male British Shorthair cat underwent colonoscopy for investigation of chronic gastrointestinal signs and persistent tenesmus. During endoscopic evaluation of a distal colonic stricture, the cat developed sudden tachycardia, severe hypoxaemia, and a dramatic decline in end-tidal carbon dioxide (EtCO_2_), accompanied by diffuse subcutaneous emphysema. Intestinal perforation was suspected, and venous blood gas analysis revealed a markedly enlarged EtCO_2_–PvCO_2_ gradient, consistent with severe ventilation–perfusion mismatch and contributing to a presumptive diagnosis of pulmonary air embolism. Anaesthetic management focused on ventilatory adjustments, including an increased respiratory rate and initially high, then moderate peak inspiratory pressures, aimed at reducing EtCO_2_ and improving oxygenation. However, improvements in oxygen saturation were limited and the cat was euthanised. Contrast-enhanced post-mortem computed tomography confirmed a distal colonic perforation with extensive air dissemination, including pneumoretroperitoneum, pneumomediastinum, pneumothorax, and subcutaneous emphysema.

## Simple Summary

Colonoscopy is commonly used in veterinary medicine to investigate diseases of the large intestine and is generally considered a low-risk procedure. However, rare complications can occur and may become life-threatening. This report describes a 15-year-old British Shorthair cat that underwent colonoscopy because of chronic gastrointestinal problems and difficulty passing faeces. During the procedure, the cat suddenly developed severe breathing and circulation problems, along with widespread swelling caused by air trapped under the skin. Anaesthetic management focused on adjusting ventilation to improve oxygenation, but the cat’s condition continued to deteriorate and euthanasia was elected. Post-mortem imaging confirmed a tear in the colon with extensive air accumulation in several body compartments.

## 1. Introduction

Colonoscopy is a commonly used procedure in veterinary medicine for diagnosing diseases of the large intestine and the rectum and, less frequently, for therapeutic interventions. It is generally considered a low-risk procedure, with complications reported as rare in both veterinary and human literature [1,2,3]. In human medicine, serious complications occur in approximately 2.8 per 1000 procedures, though this figure includes routine screening colonoscopies [4]. In dogs, serious complications associated with colonoscopy are uncommon but appear to occur at a higher rate than in humans. One study reported an overall complication rate of 0.85% [2], including regurgitation, aspiration pneumonia, and severe haemorrhage, with colonic perforation identified in 1 out of 355 dogs (0.28%) undergoing diagnostic endoscopy [2]. In cats, colonic perforation associated with colonoscopy has not been specifically reported. However, perforations involving the stomach and duodenum during gastrointestinal endoscopy have been described more frequently than in dogs, with a reported incidence of 1.6% [5].

Whilst colonoscopies in the human medical field are commonly performed awake or under sedation, general anaesthesia is most commonly utilised in animals. As such, anaesthetic-, patient-, and procedure-related factors may all contribute to the development of complications. Cardiopulmonary complications, including hypoxaemia, hypotension and arrhythmias, have been described in the literature [2,3]. Their occurrence correlates positively with higher American Society of Anaesthesiologists (ASA) patient scores and appears to be more closely associated with anaesthetic management than with the endoscopic procedure itself [2,3]. Intestinal perforation may permit insufflated air to escape into surrounding body compartments. In cats, this has been associated with the development of pneumoperitoneum and visible abdominal distention [5].

As colonic perforation is a rare complication, its anaesthetic management and associated anaesthetic risks in cats remain largely undescribed. This case report describes the anaesthetic implications and management of a cat undergoing colonoscopy that developed life-threatening complications secondary to colonic perforation.

## 2. Case Description

A 15-year-old neutered male British Shorthair cat (4.9 kg, BCS 7/9) presented to the internal medicine department of a veterinary teaching hospital for investigation of anorexia, persistent tenesmus, lethargy and signs of acute deterioration. The cat had a history of gastrointestinal disease, including triaditis and hepatic lipidosis, which was being managed on an outpatient basis.

On presentation, the cat had a grade II/VI parasternal systolic heart murmur and was estimated to be ~5% dehydrated. All other vital parameters were within normal limits. Laboratory testing revealed a moderate non-regenerative anaemia and mild azotaemia (Supplementary File S1). The cat was sedated with buprenorphine 0.015 mg/kg (Bupaq P 0.3 mg/mL, Streuli Pharma AG, Uznach, Switzerland) and alfaxalone 1 mg/kg (10 mg/mL, Graeub AG, Bern, Switzerland) intramuscularly to facilitate intravenous catheter placement and point-of-care ultrasound, which was unremarkable. Abdominal palpation revealed firm faecal matter within the colon, and subsequent abdominal radiographs confirmed moderate constipation. Abdominal ultrasound performed by a board-certified radiologist did not identify a specific underlying cause. Due to persistent clinical concern, colonoscopy was recommended, and the patient was transferred to the anaesthesia service.

As the patient was already sedated and receiving supplemental oxygen, general anaesthesia was induced using alfaxalone 1.5 mg/kg and ketamine 1 mg/kg (100 mg/mL, Streuli AG, Zürich, Switzerland) intravenously (IV), and the trachea was intubated using an appropriately sized endotracheal tube (4.0 mm ID). Anaesthesia was maintained with isoflurane (EtIso 0.77–0.91%) in an O_2_/air mixture (initially 0.6 L/min, reduced to 0.2 L/min), and Ringer’s acetate (Fresenius Kabi, Kriens, Switzerland) was administered at 3 mL/kg/h via an infusion pump. Continuous monitoring included heart rate, respiratory rate, non-invasive blood pressure, pulse-oximetry (SpO_2_), electrocardiography (ECG), capnography, fraction of inspired oxygen, and end-tidal isoflurane concentration.

**Figure 1:**
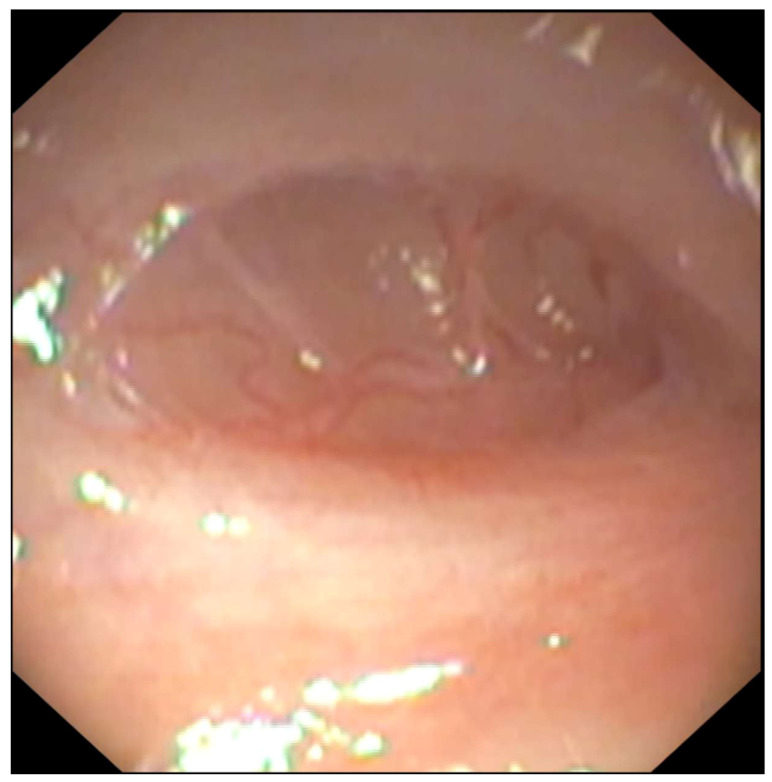
Endoscopic image following mucosal dissection after identification of the stricture.

**Figure 2:**
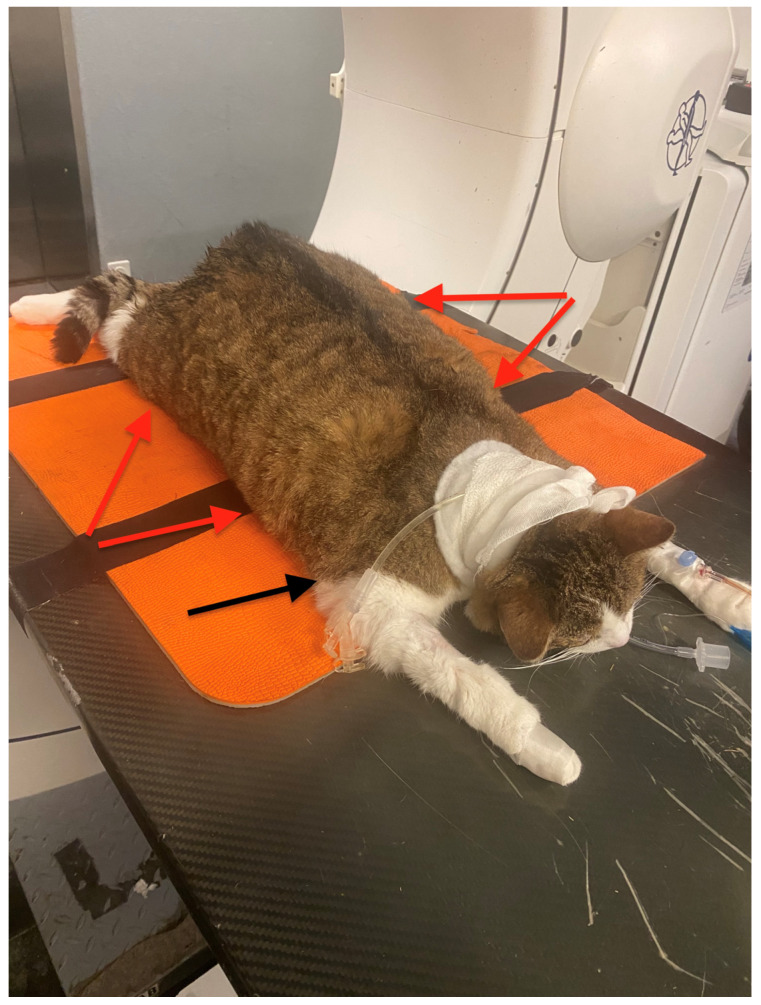
Diffuse subcutaneous emphysema following colonic perforation and insufflation during colonoscopy. Red arrows indicate generalised subcutaneous emphysema. The black arrow highlights marked proximal forelimb distension, while the distal forelimb remains of normal appearance.

During colonoscopy, the endoscope (Olympus^®^ GIF-XP160 (length 2 m, diameter 5.9 mm, work channel 2.9 mm) (Olympus Optical Co., Ltd., Tokyo, Japan) could not be advanced more than 1 cm beyond the anus due to a severe colonic stricture. Despite adequate insufflation, the colonic lumen could not be identified beyond the stricture. To facilitate passage of a dilatation balloon, careful dissection of the colonic mucosa within the stricture was attempted using biopsy forceps (Figure 1). As the colonic mucosa appeared macroscopically normal, biopsies were not obtained. After approximately 50 min of an uneventful procedure, the patient developed a sudden increase in heart rate (110 bpm to 150 bpm). A fentanyl bolus (3 ug/kg) was administered intravenously without effect. Subsequently, a marked reduction in EtCO_2_ was observed (46 mmHg to 15 mmHg). The circuit as well as the endotracheal tube were checked but EtCO_2_ remained low. At this point, diffuse and extensive subcutaneous emphysema became apparent (Figure 2).

The femoral pulse was difficult to palpate, and cardiac sounds were absent on auscultation. Cardiopulmonary arrest was suspected and basic life support (BLS) was initiated. Inhalant anaesthetic delivery was discontinued, and the breathing circuit was flushed. The patient was repositioned into left lateral recumbency, and chest compressions were performed at 100–120 bpm, with manual ventilation at 6–8 breaths per minute. Initially, peak inspiratory pressures appeared elevated (20–30 cmH_2_O), as measured using an in-line pressure manometer, during manual ventilation with delivery of a normal tidal volume. After a few breaths, the peak inspiratory pressures required to ventilate seemed to normalise. After approximately 2 min, EtCO_2_ increased to 33 mmHg, the ECG revealed sinus rhythm, and a femoral pulse was palpable following resuscitative efforts. Chest compressions were discontinued, and the patient was repositioned into sternal recumbency.

Pulse oximetry readings obtained from the hind paw were 65%, and mucous membranes appeared markedly pale. The heart rate was 180 to 200 bpm, and no pulse deficits were observed. Non-invasive blood pressure measured 120/50 mmHg (MAP: 83 mmHg), and EtCO_2_ was 47 mmHg. During this time, the patient also exhibited a gasping breathing pattern. Thoracocentesis was attempted using a butterfly catheter placed in the caudodorsal left hemithorax, but no free air could be harvested. Pulmonary embolism was considered a differential diagnosis. Ventilatory settings were adjusted to 14 to 18 bpm for respiratory rate and to 10 to 15 cmH_2_O for peak inspiratory pressures. This resulted in EtCO_2_ of 30 mmHg.

Steadily, SpO_2_ values improved to 87%, and the gasping breathing pattern ceased. The patient began exhibiting signs of superficial depth of anaesthesia (ear twitching, swallowing). Alfaxalone was administered as a bolus to maintain intubation. A venous blood gas sample was collected from the cephalic vein. Venous blood gas analysis revealed a markedly elevated PvCO_2_ of 80.1 mmHg and severe acidaemia, consistent with a mixed respiratory and metabolic acidosis, accompanied by hyperglycaemia and hyperlactatemia (Table 1).

Based on clinical signs, colonic perforation with secondary pneumoretroperitoneum, pneumomediastinum, and subcutaneous emphysema was suspected, with concurrent concern for pulmonary air embolism (PAE). Given the extent of complications and guarded prognosis, humane euthanasia was elected. A visual representation of the chronological sequence of events is provided in Supplementary File S2.

Post-mortem computed tomography (CT) with colonic contrast injection, as well as post-mortem examination, demonstrated extensive pneumoretroperitoneum with concurrent pneumomediastinum, pneumothorax, and subcutaneous emphysema from a distal colonic laceration.

## 3. Discussion

This case describes a rare but catastrophic complication during colonoscopy, a procedure generally regarded as low-risk in dogs and cats. Although colonic perforation is uncommon, it may lead to devastating consequences, as demonstrated in the case described here, resulting in severe cardiopulmonary compromise.

Previous reports in cats have typically associated colonic perforations with isolated pneumoperitoneum and abdominal distension [5]. However, depending on the location and extent of the perforation, insufflated air may track into multiple anatomical compartments, resulting in variable clinical presentations. In the present case, extensive air accumulation was identified within the subcutaneous tissues, mediastinum, thoracic cavity, and retroperitoneal space. In human medical literature, although rare, similarly extensive patterns of air tracking have been described following colon perforation [6,7,8].

Based on the postmortem CT findings, it was postulated that air passed subcutaneously, as well as into the retroperitoneal space, before tracking cranially into the mediastinum, resulting in pneumomediastinum. Although pneumothorax was identified on postmortem imaging, exploratory thoracocentesis performed during anaesthesia did not yield free air. Pneumothorax may therefore have developed later in the clinical course or postmortem. Potential mechanisms include rupture of the mediastinal pleura secondary to elevated mediastinal pressures or air migration through diaphragmatic fenestrations under increasing insufflation pressures [6,7,8]. The absence of free air during thoracocentesis, combined with the subsequent achievement of acceptable tidal volumes at conventional ventilatory pressures, further supports the possibility that clinically significant pneumothorax was not initially present. Regardless of the exact sequence of events, extensive migration of air between body compartments created substantial anaesthetic challenges, particularly with respect to maintaining cardiopulmonary stability and optimising ventilation.

Tachycardia was noted prior to the patient’s acute deterioration. Nociception was suspected and fentanyl was administered; however, the tachycardia persisted. Before further intervention could be undertaken, EtCO_2_ decreased abruptly. Following exclusion of anaesthetic machine and airway-related causes, and in the absence of palpable femoral pulses or audible cardiac sounds, cardiopulmonary resuscitation (CPR) was initiated. The decision to commence chest compressions may be considered controversial, as it was unclear whether the cat was truly in cardiopulmonary arrest. Standard criteria for CPR initiation, such as unresponsiveness and apnoea, are difficult to apply in anaesthetised patients, and clear guidelines for recognising cardiopulmonary arrest during general anaesthesia are lacking. In this case, the sudden decline in EtCO_2_ was interpreted as evidence of cardiac arrest. Interpretation of the ECG at that moment was inconclusive and artefacts/insufficient electrode contact were suspected. Based on the absence of femoral pulses and cardiac sounds, cardiopulmonary arrest was suspected. However, severe subcutaneous emphysema may have impaired ECG signal, pulse palpation and cardiac auscultation. Given that delays in CPR initiation can adversely affect outcome when cardiac arrest is present [9], prompt commencement of basic life support was considered appropriate despite the diagnostic uncertainty.

Pulmonary embolism was another differential diagnosis for the sudden decrease in EtCO_2_. Venous blood gas analysis revealed a marked disparity between EtCO_2_ (32 mmHg) and PvCO_2_ (80.1 mmHg). Ventilation–perfusion (V/Q) mismatch, resulting in increased alveolar dead space, may have been an important reason for the disparity. Potential causes included profound hypotension with impaired pulmonary perfusion, tension pneumomediastinum, inadvertent endobronchial intubation, or pulmonary embolism secondary to thrombus or air emboli [10]. Acute respiratory distress syndrome could also be considered but is very unlikely given the clinical course. In dogs, experimental models have demonstrated PAE following induced pneumomediastinum [11]. Profound hypotension is also a plausible contributing factor, as it may independently reduce pulmonary blood flow and increase dead space ventilation, thereby exaggerating the observed EtCO_2_–PvCO_2_ gradient [10].

In this case, the extensive distribution of air may support a presumptive diagnosis of PAE. The abrupt decline in EtCO_2_ and concurrent desaturation further supports this suspicion, as both are recognised features of PAE [12,13]. The severity of clinical signs depends on both the volume and rate of air entrainment [13]. Both are presumed to be significant in this case given the cat’s rapid and dramatic deterioration. However, these findings are non-specific and may also occur with severe cardiovascular collapse or other causes of acute reduction in pulmonary blood flow. Definitive diagnosis of PAE generally requires advanced diagnostic modalities such as trans-oesophageal echocardiography, precordial Doppler ultrasonography, or angiography [13,14]. None of which were available in this case; therefore, the diagnosis remains presumptive.

The marked tachycardia observed prior to acute decompensation may reflect several physiological processes, including nociception, sympathetic activation, hypovolaemia, or early cardiovascular compromise. Although non-specific, tachycardia may also occur in the setting of PAE. Pulmonary embolism-induced increases in pulmonary vascular resistance and right ventricular wall tension prompt reflex increases in heart rate and contractility to preserve cardiac output [15]. It is possible that the initial tachycardia observed in this case may have been related to early air emboli. Furthermore, tachycardia is recognised as a predictor of adverse outcome in pulmonary embolic disease [15].

In response to persistent oxygen desaturation and the widened EtCO_2_-PvCO_2_ gradient, ventilatory adjustments were implemented. During the initial ventilation strategy, consisting of 6–8 bpm at 8–10 cmH_2_O, SpO_2_ remained low. Considering the presumptive presence of PAE, reliance on EtCO_2_ as a marker of adequate ventilation may have underestimated arterial carbon dioxide concentrations due to increased alveolar dead space, resulting in significant hypercapnia. Increasing both respiratory rate (14–18 breaths/min) and inspiratory pressure (10–15 cmH_2_O), targeting a lower EtCO_2_, resulted in gradual improvement in haemoglobin oxygen saturation. Despite increasing minute volume, the improvement in haemoglobin oxygen saturation was gradual and slow. This may have been due to persistent dead space ventilation secondary to the suspected embolism, which may only gradually have resolved over time. In the medical literature, treatment options for PAE include aspiration using thrombus aspiration catheters, hyperbaric oxygen therapy, and extracorporeal membrane oxygenation [12]. Forceful saline injection to fragment and disperse air bubbles distally has also been described as a more accessible intervention [14], and may have been a more practical consideration in this setting.

Ventilation may have been further complicated by pneumomediastinum and the suspected development of pneumothorax, particularly as the latter may result in alveolar collapse, atelectasis and impaired oxygenation. In such situations, limiting tidal volumes and plateau pressures while minimising positive end-expiratory pressure is generally recommended [16].

Ultimately, the patient was euthanised due to the poor prognosis associated with both the anaesthetic complications and the underlying rectal stricture. Severe ventilation-perfusion mismatch, together with suspected bacterial translocation following colonic perforation, markedly worsened the prognosis. Although supportive strategies such as prolonged ventilation and the placement of subcutaneous or intrapleural drains might have provided temporary stabilisation, the introduction of contaminated gas into multiple body compartments significantly increased the risk of widespread septic complications. Septic peritonitis is a well-recognised consequence of gastrointestinal perforation [17], and in this case, bacterial contamination likely extended into the mediastinum and pleural space, further compounding the poor prognosis.

During gastrointestinal endoscopy, anaesthetists should remain vigilant for this rare but severe complication. Procedural risk factors, particularly severe colonic strictures, should be recognised as they increase technical complexity. In the present case, the stricture was too narrow to permit balloon dilatation, necessitating blind mucosal dissection, in which inadvertent laceration of the lateral colonic wall is a recognised risk. Insufflation pressure was not recorded and therefore cannot be excluded as contributing to the extent of air extravasation. Although carbon dioxide insufflation is not routinely used in veterinary endoscopy, its use may theoretically reduce the risk of overdistension [18]. Finally, if subcutaneous emphysema is detected, gastrointestinal perforation with air leakage should be suspected and insufflation should be immediately discontinued. In the event of a sudden decrease in EtCO_2_, prompt assessment of airway and endotracheal tube patency, confirmation of capnography and circuit function, and rapid evaluation for cardiopulmonary arrest should be undertaken without delay.

## 4. Conclusions

In this case, colonic perforation during colonoscopy led to extensive gas dissemination, including subcutaneous emphysema, pneumoretroperitoneum, pneumomediastinum and eventually pneumothorax. Early recognition of, and prompt response to, sudden cardiopulmonary changes may improve outcomes in similar cases. This report emphasises the importance of maintaining anaesthetic vigilance during seemingly benign procedures and highlights the value of pre-anaesthetic procedural risk discussions.

## Figures and Tables

**Table 1:** Venous blood gas and electrolyte analyses during the critical incident following resuscitative efforts. The sample was collected from the right cephalic vein, placed directly into a heparinized syringe and analysed immediately (RAPIDPoint^®^ 500, Siemens Healthineers (Erlangen, Germany)). EtCO_2_ at the time of sample collection was 32 mmHg. Values outside reference intervals are in bold.

Parameter	Value	Reference
pH	**7.00**	7.35–7.45
PvCO_2_ (mmHg)	**80.1**	28.6–44.7
HCO_3_^−^ (mmol/L)	**19.3**	19.7–24.8
Base Excess (mmol/L)	**−13.5**	−6.7–1.5
Na^+^ (mmol/L)	**152.3**	143.7–151.1
K^+^ (mmol/L)	4.35	3.66–4.72
iCa^2+^ (mmol/L)	1.32	1.23–1.4
Cl^−^ (mmol/L)	**118**	109–117
Anion Gap (mmol/L)	19.3	11.6–21.2
Glucose (mmol/L)	**16.1**	3.9–6.4
Lactate (mmol/L)	**3.73**	0.43–2.1

## Data Availability

The original contributions presented in this case are included in the article/Supplementary Material. Further inquiries can be directed to the corresponding author.

## Supplementary Materials

The following supporting information can be downloaded at: https://www.mdpi.com/article/10.3390/vetsci13070707/s1, File S1 Pre-anaesthetic haematology & biochemistry; File S2 Chronological sequence of events.

## Abbreviations

The following abbreviations are used in this manuscript:

CPR	Cardiopulmonary Resuscitation
EtCO_2_	End-tidal Carbon Dioxide
MAP	Mean Arterial Pressure
PAE	Pulmonary Air Embolism
