# Supplementary material for: Anaesthetic Challenges During Colonoscopy-Induced Intestinal Perforation in a Cat
Source: Vet Sci. 2026 Jul 19;13(7):707. doi: 10.3390/vetsci13070707 (PMC13431627; doi:10.3390/vetsci13070707)
Supplement: Supplementary file 1 [file vetsci-13-00707-s001.zip › Supplementary File S1 Pre-anaesthetic haematology & biochemistry.pdf]

Supplementary File S1: Pre-anaesthetic haematology & biochemistry

Parameter	Result	Reference range	Unit
**Hct**	0.25	0.32–0.48	L/L
**Erythrocytes**	5.46	7.08–11.15	x10^12/L
**Hb total**	85	111–169	g/L
**MCV**	46	38–50	fL
**MCH**	16	13–17	pg
**MCHC**	336	325–359	g/L
**RDW**	19.8	14.0–17.3	%
**Reticulocytes**	16	9–70	x10^9/L
**Reticulocytes %**	0.3	0.1–0.8	%
**CHr**	20.3	15.7–21.9	pg
**MCVr**	68.4	51.2–74.9	fL
**Thrombocytes**	549	180–520	x10^9/L
**MPV**	13.2	9.0–22.1	fL
**Leukocytes**	13.31	3.78–15.6	x10^9/L

Cell type	Result	Reference range	Unit
**Nucleated RBCs %**	0.0	0.0–0.5	per 100 leukocytes
**Band neutrophils %**	0.5	0.0–1.5	%
**Segmented neutrophils %**	79.0	28.0–78.0	%
**Lymphocytes %**	16.5	15.5–63.0	%
**Monocytes %**	3.0	0.0–7.0	%
**Eosinophils %**	1.0	1.0–16.0	%
**Nucleated RBCs**	0.00	0.0–0.03	x10^9/L

Cell type	Result	Reference range	Unit
**Band neutrophils**	0.07	0–0.1	x10^9/L
**Segmented neutrophils**	10.51	1.6–8.5	x10^9/L
**Lymphocytes**	2.20	1.1–7.2	x10^9/L
**Monocytes**	0.40	0.0–0.8	x10^9/L
**Eosinophils**	0.13	0.1–1.4	x10^9/L
**Basophils**	0.00	0.0–0.1	x10^9/L

Test	Result	Reference range	Unit
**Icterus Index**	0		
**Lipemia Index**	6		
**Na**	151	147–157	mmol/L
**K**	3.90	3.26–5.11	mmol/L
**Cl**	117	113–123	mmol/L
**Ca**	2.39	2.34–2.88	mmol/L
**P**	1.50	0.77–1.89	mmol/L
**Glucose**	5.74	3.22–9.86	mmol/L
**Cholesterol**	2.34	1.95–7.18	mmol/L
**Triglyceride**	0.30	0.23–1.45	mmol/L
**Total protein**	68.5	63.3–83.2	g/L
**Albumin**	30.3	30.0–43.3	g/L
**Globulin**	38.2	25.8–50.6	g/L
**Urea**	11.9	6.5–13.5	mmol/L
**Creatinine**	220	64–163	µmol/L
**SDMA**	34	<14	µg/dL
**Bilirubin**	0.8	0.0–2.2	µmol/L

[OFFICIAL]

Test	Result	Reference range	Unit
**ALAT (GPT)**	91	25–87	U/L
**AP**	21	13–69	U/L
**ASAT (GOT)**	26	12–40	U/L
**CK**	1692	68–555	U/L
**gGT**	0	<1	U/L
**GLDH**	3	0–5	U/L
**Lipase (DGGR)**	15	8–39	U/L
**Serum Amyloid A (SAA)**	5.7	<5.2	mg/L

[OFFICIAL]
