# Supplementary material for: Anaesthetic Challenges During Colonoscopy-Induced Intestinal Perforation in a Cat
Source: Vet Sci. 2026 Jul 19;13(7):707. doi: 10.3390/vetsci13070707 (PMC13431627; doi:10.3390/vetsci13070707)
Supplement: Supplementary file 1 [file vetsci-13-00707-s001.zip › Supplementary File S2 Chronological sequence of events.pdf]

Supplementary File S2: Chronological sequence of events (T0 = induction; times in minutes; TI = intervention periods). Exact event timing is approximate due to the emergent clinical context; sequence reflects observed progression.

T0: Patient Induction

T15: Colonoscopy started

T50: Tachycardia

Heart rate increased from 110 to 150 bpm, fentanyl administration did not resolve the tachycardia.

T55: Sudden decline in $EtCO_2$

$EtCO_2$ decreased from 36 to 15 mmHg. Extensive subcutaneous emphysema was promptly observed.

TI: Initiation of BLS

Femoral pulse was difficult to palpate and cardiac sounds were difficult to auscultate. BLS consisted of chest compressions and manual ventilation.

TI: Return of spontaneous circulation

$EtCO_2$ increased to 33 mmHg, the ECG revealed sinus rhythm, and a femoral pulse was palpable following resuscitative efforts.

TI: Ventilator adjustments

Consistent hypoxaemia, based on decreased SpO2 prompted adjustments in ventilatory settings.

TI: Venous blood gas sampling

Venous blood gas analysis revealed a marked respiratory acidosis, and a wide $EtCO_2$-P_vCO_2 difference.

T90: Euthanasia

The patient was euthanised given the extent of complications and guarded prognosis.

[OFFICIAL]
